# Non-coding RNAs including miRNAs, piRNAs, and tRNAs in human cancer

**DOI:** 10.18632/oncotarget.5048

**Published:** 2015-07-31

**Authors:** Mieke Heyns, Olga Kovalchuk

**Affiliations:** ^1^ Department of Biological Sciences, University of Lethbridge, Lethbridge, Canada

**Keywords:** cancer, non-coding RNA, miRNA, piRNA, tRNA

## Abstract

Over 98% of our genes code for RNA transcripts that will never become translated into protein. Numerous non-coding RNA (ncRNA) transcripts are structurally and functionally diverse. In particular, micro RNAs (miRNAs), piwi-interacting RNAs (piRNAs), and, more recently, transfer RNAs (tRNAs) are implicated as regulators of key genes and processes that are involved in various human diseases, including cancer. Here, we summarize the recent findings and perspectives in the small RNA and cancer research.

Breakthroughs in non-coding RNA biology, including the sequencing of the human genome, reveal that over 98% of our genes code for RNA transcripts that will never become translated into protein [1, 2]. These non-coding RNA (ncRNA) transcripts are very diverse in both structure and repertoire of biological function [3]. ncRNAs fulfill an ever-increasing range of functions as knowledge about these RNAs grow, and some of these functions include the control of cell differentiation, proliferation, apoptosis, stress response, and genome stability; therefore, epigenetic and genetic defects in ncRNAs and their associated processing machinery are common hallmarks of cancer and other human diseases [4-6]. In particular, micro RNAs (miRNAs), piwi-interacting RNAs (piRNAs), and, more recently, transfer RNAs (tRNAs) are implicated as regulators of key developmental genes with important involvement in human diseases, such as birth defects and cancer [7–9].

These ncRNAs have all been found to be deregulated in cancer, with miRNA being profoundly better characterized than the other subclasses. Genetic alterations caused by chromosomal abnormalities leading to deletion, amplification, or rearrangements are especially prevalent in the more than 50% of miRNAs located near fragile genome sites [10]. Recent research has also highlighted the importance of epigenetic effects on miRNA, with 10% of miRNAs regulated by cytosine DNA methylation [11]. The role of miRNAs as trans-acting factors that suppress translation or induce messenger RNA (mRNA) degradation of target genes has naturally categorized these ncRNAs into two groups, namely, oncogenic miRNAs (oncomiRs) and tumour suppressor miRNAs. Numerous studies have considered the relationship between miRNAs and their target(s) with the hallmarks of cancer, including self-sufficient cell differentiation, proliferation, and evasion of apoptosis as well as responses to carcinogens [12-15]. Although these studies emphasize the potential therapeutic effects of miRNAs, perhaps the next advances in research will involve the potential of miRNAs to act as biomarkers for disease detection and progression, which will advance individualized treatments [16-18]. Much recent research has focused on the non-invasive approach of using circulating miRNA in the serum as markers for disease progression, and the findings can potentially be integrated with therapeutic treatments in the future [19-21].

As their name suggests, piRNAs complex with PIWI proteins to form piRISC as the effector complex in retrotransposon silencing within the germline [8]. PiRNAs have been implicated in testicular cancers, as well as other cancer types, but their exact role in tumourigenesis remains elusive [22, 23]. PIWI proteins are better understood than piRNAs, with direct examples of overexpression in a variety of germline and somatic tumours, including testicular, ovarian, endometrial, prostate, breast, and gastrointestinal cancers in humans [24-27]. Specifically in ovarian cancer, PIWIL2 overexpression has been linked to resistance to the chemotherapy drug cisplatin through increased chromatin condensation preventing access by DNA repair machinery [28]. Interestingly, PIWIL2 is shown to form immune complexes with piR-932 and be upregulated in breast cancer stem cells with a possible role in methylating latexin, a tumour suppressor gene that reduces the risk of senesced stem cells transitioning to cancer stem cells [29]. With the previously established evidence on PIWI protein deregulation in cancer, the potential that piRNA is also aberrantly expressed in a variety of cancer types is very likely because it is a vital part of the piRISC effector complex that allows the recognition of targets. This area is a newly emerging topic of study in cancer research and will certainly grow immensely in the future.

Increased cell proliferation requires elevated protein synthesis levels, which makes the correlation between tumour cells and deregulated tRNAs, a component in translation, probable. With limited research, a few studies have demonstrated that the components involved in translation are shown to be dysregulated in cancer, including elevation of tRNA transcripts in ovarian cancer [30]. With few studies using human models, Marshall *et al*. was able to show the direct correlation between overexpression of the initiator tRNA (iMET) and increased cell proliferation, which leads to oncogenic tranformation of fibroblasts and thus tumour formation in mice; this finding demonstrates that tRNA deregulation was not just a byproduct of cancer but a driving force in carcinogenesis [31]. The underlying mechanism is not clear but likely pertains to the direct role of tRNAs in controlling the speed of translation elongation of various oncogenes [32]. Therefore, specific tRNA levels may be important in speeding up the translation process of key genes required for tumourigenesis, as well as cancer development and progression. The first genome-wide tRNA expression study to look at all tRNAs showed that compared with normal breast tissue, the breast cancer tumour, had up to a 10-fold increase in nuclear- and mitochondrial- encoded tRNAs [33]. The same study noted the potential of tRNAs as molecular signatures for the diagnosis of tumour type and disease progression, but also noted tRNA levels may be more simply used to distinguish cancerous from non-cancerous tissues. Mirroring recent breakthroughs in miRNA detection, tRNA halves, previously thought to be a by-product of tRNA degradation, may potentially serve as a novel type of blood-based marker for cancer detection and monitoring. Using deep sequencing, a recent study showed that patients with breast cancer had significant increases in 5′ tRNA halves derived from specific tRNAs, as well as decreased 5′ tRNA halves from other specific tRNAs linked to certain disease characteristics [34]. These findings suggest the association between breast cancer and changes in novel circulating RNA species reflect some aspects of the biology of the tumour. In the future, these research efforts will lead to an increased knowledge base about these non-coding RNAs, which will hopefully translate into effective care for cancer patients.
